# Screw fixation without fusion for low lumbar chance fracture accompanied by spinal epidural hematoma in patient with ankylosing spondylitis

**DOI:** 10.1186/s12891-023-06428-4

**Published:** 2023-04-24

**Authors:** Dae Kyun Kim, Seok Won Kim

**Affiliations:** Department of Neurosurgery, College of Medicine, Chosun University, 365 Pilmun-daero, Dong-gu, Gwangju, 61453 Republic of Korea

**Keywords:** Ankylosing spondylitis, Chance fracture, Epidural hematoma

## Abstract

Ankylosing spondylitis (AS) is a chronic inflammatory disease involving the sacroiliac joint and axial spine. AS may render the ankylosed spine prone to trauma and cause an increased frequency of associated epidural hematomas in spine fractures. Herein, we report a rare case of L5 chance fracture and epidural hematoma in a 27-year-old female patient with AS. She was treated surgically but without bone fusion or decompressive laminectomy due to the neurologically intact status despite significant neural compression by the spinal epidural hematoma (SEH). We believe that conservative treatment with close observation of neurological status may be effective in SEH presenting with mild neurological symptoms despite significant neural compression.

## Introduction

Ankylosing spondylitis (AS) is a chronic inflammatory disease of the sacroiliac joint and axial spine that is closely linked to human leukocyte antigen (HLA)-B27 [1]. Chronic inflammation, bone remodeling, and osseous resorption lead to osteoporosis and a more rigid spine that predisposes patients to increased fractures, which can be detected on radiological studies. The lower lumbar spine is deeply located in flexible segments and has physiological lordosis. Therefore, Chance fractures of the lower lumbar spine are uncommon injuries compared to those of the cervical or thoracolumbar junction, even in patients with AS. Spinal epidural hematoma (SEH) in spine fractures in patients with AS may occur and have dire neurologic deficits if not treated urgently [2]. This paper describes a 27-year-old patient with AS and a L5 Chance fracture accompanied by spinal SEH. It was possible to preserve the motion segments without decompressive laminectomy or conventional long-level fusion. To our knowledge, no studies have specifically focused on short-segment fixation without fusion for low lumbar fractures accompanying SEH in patients with AS.

## Case report

**Fig. 1:**
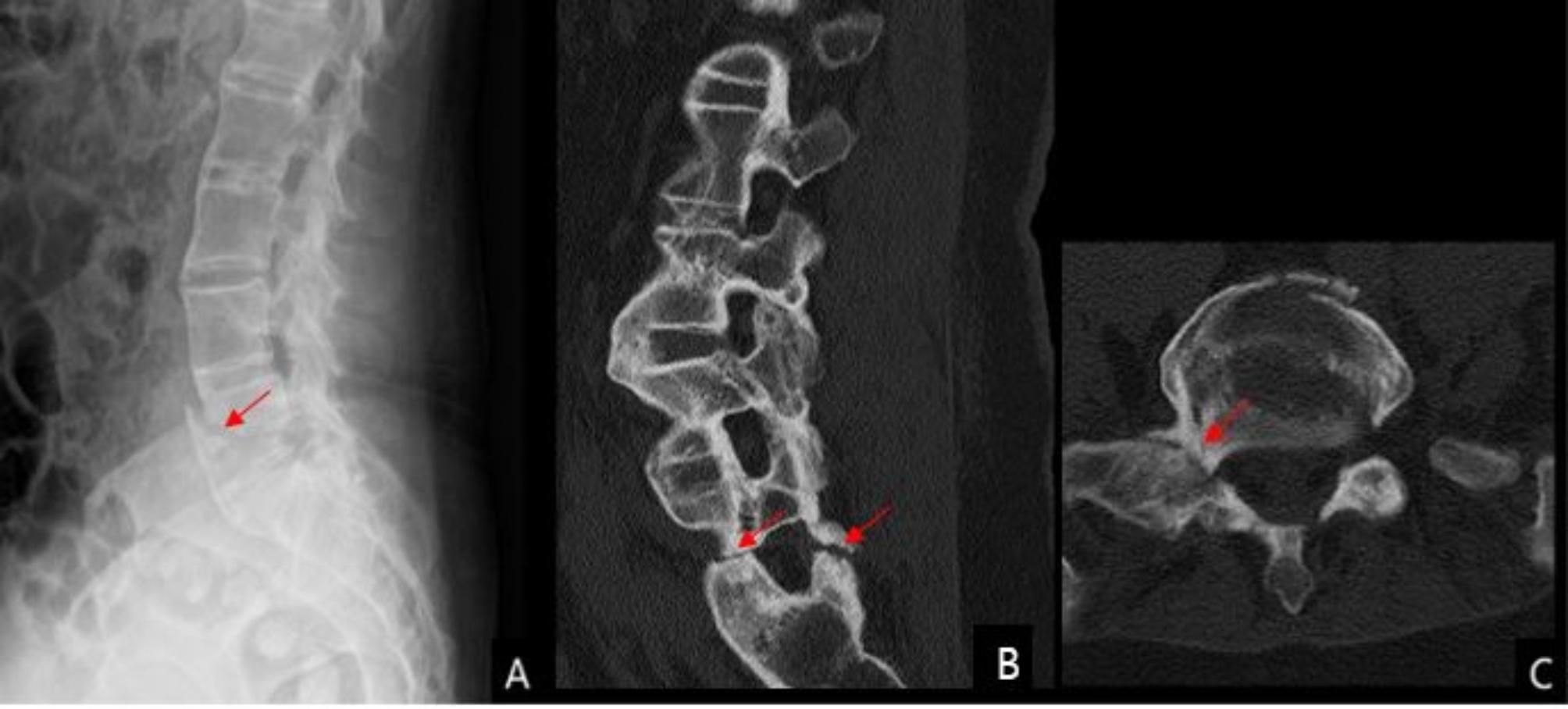
**A**) Simple lateral radiograph reveals discontinuity of ossified ligament and displaced L5 fracture. **B** & **C**) Computed tomography (CT) scans show vertebral body and posterior element fractures (arrows)

**Fig. 2:**
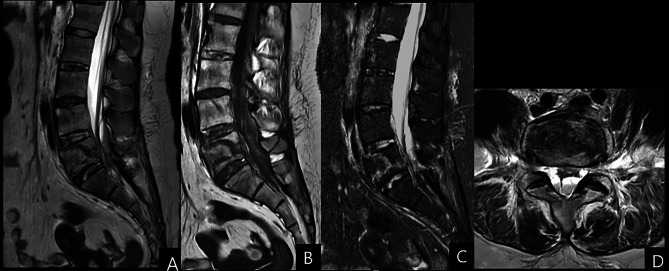
**A**) T2-weighted sagittal, **B**) T1-weighted sagittal, **C**) fat-suppressed sagittal and **D**) T2-weighted axial MR images show epidural hematoma extending from L4 to S1 in the posterior part of epidural space

**Fig. 3:**
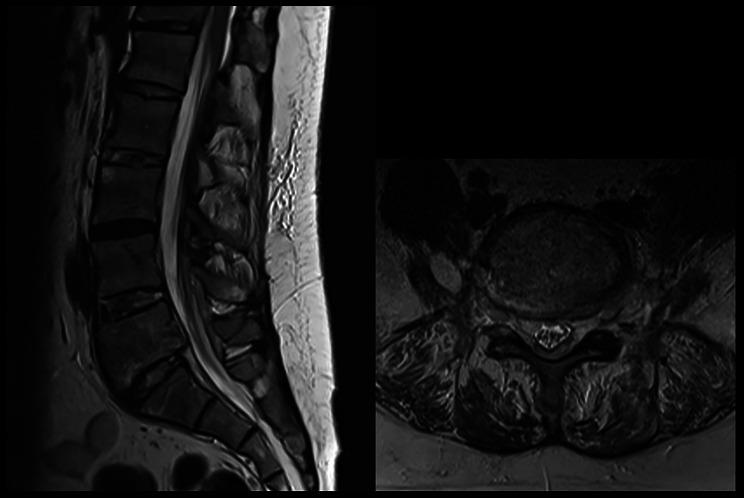
T2-weighted magnetic resonance (MR) images taken six days after injury show spontaneous regression of hematoma

**Fig. 4:**
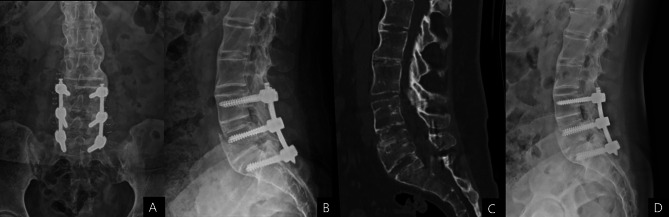
**A** & **B**) Simple radiographs show short-segment fixation without fusion by percutaneous technique. **C** & **D**) Computed tomography scan and lateral radiograph at 8 months postoperative reveal bony union of L5

A 27-year-old female patient with a 5-year history of AS was admitted to the emergency room (ER) with severe low back pain (LBP) radiating to both lower extremities. She had a history of accidental fall from chair on the same day. She had been treated with selective Cox-2 inhibitor and TNF-α antagonist to alleviate pain and to recover physical functions for 2 years before accident. Medical treatment was continued through consultation with rheumatologist. Despite severe LBP and radiating pain, neurological examination revealed no bilateral motor or sensory deficits in the legs. The deep tendon reflexes were normoreactive, and there were no neurological abnormalities related to cauda equina syndrome such as bladder or bowel dysfunction. Simple radiography and computed tomography (CT) revealed a discontinued ossified paraspinal ligament and Chance fracture of the L5 vertebra extending into the posterior elements (Fig. 1). Magnetic resonance (MR) images of the lumbar spine showed a displaced fracture of L5, associated with a hematoma in the posterior part of the epidural space from L4 to S1 (Fig. 2). Emergent hematoma removal and long-level screw fixation were planned initially, but were canceled due to the absence of motor and sensory deficits except for LBP and radiating pain in both lower extremities. Close observations and medical treatments were designed for SEH. Follow-up MRI performed six days later showed almost complete resolution of the hematoma, and the radiating pain in both lower extremities disappeared (Fig. 3). We performed percutaneous short-segment fixation without bone fusion and decompressive laminectomy due to the absence of neurological deficits. She was allowed to ambulate in a TLSO brace on the third postoperative day that was applied for three months after screw fixation (Fig. 4). The patient was able to walk independently without any difficulties. There were no neurological deficits related to cauda equina or radiculopathy during the eight postoperative months of follow-up.

## Discussion

AS is an inflammatory rheumatic disease involving the bones and ligaments of the spine. The most characteristic features of AS are syndesmophytes and ankylosis, resulting in ossification of the entire spine. Another important feature is low bone mineral density as a result of chronic inflammation and diffuse atrophy [3]. These two clinical features of AS not only increase the risk of vertebral fractures but also add to the difficulties in treating vertebral fractures with AS. Fractures in AS may occur at any site even after trivial trauma, but most fractures are localized to the cervical, cervicothoracic, or thoracolumbar junction [4]. The lower lumbar spine is rarely affected and represents a small percentage of spine injuries even in patients with AS. The iliolumbar ligaments and their location below the pelvic brim are two stabilizing factors that are unique to these fractures compared with those of the cervical, cervicothoracic, or thoracolumbar junction. The incidence of SEH is higher in patients with AS than in healthy people [5, 6]. It is reasonable to assume that when SEH is associated with a vertebral fracture, bleeding could originate in the fractured spongy bone or from the damaged venous plexus of the epidural space with a secondary collection of blood. Ossification of the ligaments in these patients increases the risk of SEH. There is some controversy regarding the best way to manage SEH, with reports describing good results after both conservative and surgical management [7]. Emergency surgical decompression should be performed as soon as possible in cases of progressive neurological impairment to ensure neurologic recovery [8]. However, close observation and conservative treatment could be considered in cases of mild and nonprogressive neurologic impairment despite the significant size of SEH. Stabilizing surgery using long-segment fixation has been used to treat spine fractures in patients with AS. However, we performed short-segment screw fixation without decompressive laminectomy and hematoma removal in our patient. Although the SEH size was significant, spontaneous regression was confirmed after close observation and medical treatment. The advantages of short-segment fixation without fusion in this patient include immediate pain relief, elimination of donor site pain, reduced blood loss, and short operative time. Moreover, early ambulation and motion preservation are possible compared to long-level instrumentation and fusion.

## Conclusion

Lower lumbar fractures accompanying SEH are extremely rare, even in patients with AS. We believe that close observation and medical management can be an effective way to cause spontaneous regression of SEH in patients presenting with mild neurological symptoms.

## Data Availability

The datasets used and/or analysed during the current study available from the corresponding author on reasonable request.

## References

[1] Pedersen SJ, Maksymowych WP (2019). The pathogenesis of ankylosing spondylitis: an update. Curr Rheumatol Rep.

[2] Akcay Yalbuzdag Ş, Erol AM, Celik C, Solum S (2014). Ankylosing spondylitis diagnosed after epidural hematoma and paraplegia: a case report. Arch Rheumatol.

[3] An SB, Kim KN, Chin DK, Kim KS, Cho YE, Kuh SU (2014). Surgical outcomes after traumatic vertebral fractures in patients with ankylosing spondylitis. J Korean Neurosurg Soc.

[4] Westerveld LA, van Bemmel JC, Dhert WJ, Oner FC, Verlaan JJ (2014). Clinical outcome after traumatic spinal fractures in patients with ankylosing spinal disorders compared with control patients. Spine J.

[5] Jacobs WB, Fehlings MG (2008). Ankylosing spondylitis and spinal cord injury: origin, incidence, management, and avoidance. Neurosurg Focus.

[6] Hanna G, Uddin SA, Trontis A, Ross L (2021). Epidural hematoma in patients with ankylosing spondylitis requiring surgical stabilization: a single-institution retrospective review with literature analysis. Neurosurg Focus.

[7] Mohr BW, Mohr K, Hassepass U, Richter HP, Kast E (2004). Spinal hematoma unrelated to previous surgery: analysis of l5 consecutive cases treated in a single institution within a 10-year period. Spine.

[8] Lee HH, Park SC, Kim Y, Ha YS (2015). Spontaneous spinal epidural hematoma on the ventral portion of whole spinal canal: a case report. Korean J Spine.

